# Robotic Heller Myotomy for Achalasia: A Narrative Review of the State-of-the-Art Technique and Its Future Directions

**DOI:** 10.7759/cureus.95637

**Published:** 2025-10-28

**Authors:** Rezuana Tamanna, Elmoatazbellah Nasr, Ahmed Swealem, Samir Bin Halim, Momen Abdelglil, Nazeer Ibraheem, Muhammad Rakib Hasan, Shaila Mostareen, Anika Tahsin, Zaid Al-Hamid

**Affiliations:** 1 General Surgery, Craigavon Area Hospital, Northern Ireland Medical and Dental Training Agency (NIMDTA), Portadown, GBR; 2 General Surgery, Calderdale and Huddersfield National Health Service (NHS) Foundation Trust, Huddersfield, GBR; 3 Orthopedics, Southmead Hospital, North Bristol National Health Service (NHS) Foundation Trust, Bristol, GBR; 4 General Surgery, Watford General Hospital, Watford, GBR; 5 Pediatric Surgery, Mansoura University Children Hospital, Mansoura, EGY; 6 Urology, The Royal Wolverhampton National Health Service (NHS) Trust New Cross Hospital, Wolverhampton, GBR; 7 Urology, Watford General Hospital, West Hertfordshire Hospitals National Health Service (NHS) Foundation Trust, Watford, GBR; 8 Obstetrics and Gynaecology, Watford General Hospital, Watford, GBR; 9 Gastrointestinal Surgery, University Hospitals of Leicester National Health Service (NHS) Trust Leicester Royal Infirmary, Leicester, GBR; 10 School of Biosciences, The University of Sheffield, Sheffield, GBR; 11 Colorectal Surgery, Blackpool Teaching Hospitals National Health Service (NHS) Foundation Trust, Blackpool, GBR

**Keywords:** achalasia, laparoscopic heller myotomy, lhm, myotomy, peroral endoscopic myotomy, poem, rahm, robotic heller myotomy

## Abstract

Achalasia is a rare motility disorder causing impaired lower esophageal sphincter relaxation and absent peristalsis. Surgical myotomy remains key; robotic-assisted Heller myotomy (RAHM) is increasingly used alongside laparoscopic Heller myotomy (LHM) and peroral endoscopic myotomy (POEM). Evidence indicates RAHM yields outcomes similar to LHM with fewer mucosal perforations, likely due to improved visualization and dexterity. POEM offers shorter operations and longer, tailored myotomies, especially for type III but higher postoperative reflux without antireflux measures; fundoplication is standard after surgical myotomy. Long-term control is durable across approaches; cohorts suggest RAHM advantages in barium emptying, reintervention, and Eckardt scores. RAHM costs more than LHM, while POEM can be cost-efficient. Innovations may enhance clinical precision. Selection should consider anatomy, reflux risk, prior therapy, and expertise. In this review, we aimed to synthesize evidence on robotic Heller myotomy (RHM) for achalasia, comparing indications, long-term outcomes versus LHM and POEM, appraising reflux management and costs, and outlining learning curves, limitations, and future research priorities.

## Introduction and background

Achalasia is a rare primary esophageal motility disorder characterized by the inability of the lower esophageal sphincter (LES) to relax adequately during swallowing, coupled with the absence of effective esophageal peristalsis [1-3]. The global incidence of achalasia is estimated at approximately 0.78 cases per 100,000 person-years [4-6]. Achalasia is classified into three HRM subtypes according to the Chicago Classification v4.0: type I (classic, absent peristalsis with minimal pressurization), type II (absent peristalsis with panesophageal pressurization), and type III (spastic, characterized by premature/distal esophageal contractions) [5].

Management of primary idiopathic achalasia comprises non-surgical and surgical options. Non-surgical therapies include pharmacologic treatment, typically used as a bridge to definitive care or when other options are contraindicated, endoscopic botulinum toxin injection, and graded pneumatic dilation (PD). Surgical options can be organized by invasiveness; the less invasive endoscopic approach is peroral endoscopic myotomy (POEM) [3-7]. More invasive approaches include laparoscopic Heller myotomy (LHM) or robotic Heller myotomy (RHM) with partial fundoplication [8].

RHM has become more and more common over the past 10 years. LHM and RHM are equally effective; however, RHM causes fewer mucosal perforations [9]. Consequently, it is feasible that in the near future, a greater proportion of surgical myotomies will be performed robotically. On the other hand, RHM is more expensive than LHM [10-12]. Multiple systematic reviews and meta-analyses have demonstrated that RHM is at least as effective as LHM in symptom relief, with some studies reporting lower rates of intraoperative complications, particularly esophageal perforation, and shorter hospital stays, albeit with longer operative times and higher costs [8,13-15]. In this review, we aim to synthesize evidence on robotic Heller myotomy for achalasia, covering indications, patient selection, comparative efficacy versus LHM/POEM, perioperative complications, long-term outcomes, and future research directions.

## Review

Methodology

We conducted a narrative review focused on robotic Heller myotomy for achalasia and its comparators (laparoscopic Heller myotomy (LHM) and peroral endoscopic myotomy (POEM)). We searched PubMed/MEDLINE, Scopus, Web of Science, and the Cochrane Library (January 2010-September 2025) using Boolean combinations of terms related to achalasia, Heller myotomy/RAHM, LHM, POEM, outcomes (Eckardt score [10], perforation, GERD/reflux, reintervention), perioperative metrics, fundoplication, learning curve, redo surgery, and cost-effectiveness. Reference lists of key guidelines and reviews were hand-searched for additional studies. We included peer-reviewed original studies (RCTs, cohorts, case series ≥5 patients), systematic reviews/meta-analyses, and clinical guidelines that reported clinical, physiologic, or economic outcomes relevant to RAHM/LHM/POEM; we excluded editorials, conference abstracts without full text, non-English articles without reliable translation, and animal/cadaveric or purely technical reports unless directly informative to clinical practice. Two reviewers independently screened titles/abstracts and full texts, resolving disagreements by discussion, and extracted data on study design, population, interventions (myotomy technique ± fundoplication), and outcomes. Given heterogeneity across designs, subtypes, and endpoints, we undertook qualitative, thematic synthesis without meta-analysis, privileging higher-level evidence and consistency across sources.

Historical context and technology

Heller first described extramucosal cardiomyotomy in 1913; over the 20th century, treatment evolved from open surgery and pneumatic dilation to minimally invasive approaches. Laparoscopic Heller myotomy (usually paired with a partial fundoplication) became the modern standard, with wide adoption through the 1990s-2000s and durable symptom relief in most series. Narrative histories and reviews chart this progression and set the stage for robotics as a technological extension of laparoscopy [16,17].

Robotic assistance entered routine surgical practice after the da Vinci system received FDA clearance in 2000, which marked the first widely adopted, commercially successful robotic platform in human surgery. This system provided immersive three-dimensional visualization and articulated, wristed instruments that improved dexterity, tremor control, and surgeon ergonomics compared with conventional rigid laparoscopic tools. These features helped overcome several technical limitations of standard laparoscopy and accelerated clinical uptake. Over time, newer generations of robotic platforms, including the Xi system (introduced in 2014) and the single-port (SP) system (which later received U.S. clearance in 2018), further improved arm configuration, vision systems, docking efficiency, and access through fewer incisions. These advances expanded applications in foregut surgery (such as Heller myotomy) and enabled more efficient operating room workflow and team coordination [18,19].

**Figure 1:**
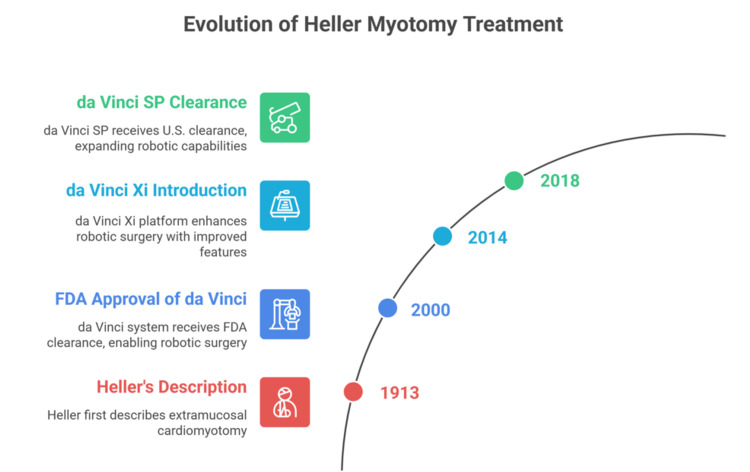
Historical context and technology of robotic-assisted Heller myotomy The da Vinci system received US FDA clearance in 2000, bringing immersive three-dimensional vision and wristed, tremor-filtered instruments into routine minimally invasive surgery. The next-generation da Vinci Xi platform, introduced in 2014, optimized arm architecture, vision, and docking to allow more versatile port placement and smoother foregut access. The da Vinci SP (single-port) system later obtained U.S. clearance in 2018, permitting a 3D camera and multi-jointed instruments to be deployed through a single incision. These stepwise advances underpin the application of robotic assistance to Heller myotomy by improving precision, exposure, and team workflow. Figure credit: Momen Abdelglil. References: [8,18-20].

Within this technological arc, RAHM has been compared with LHM in multiple analyses: contemporary meta-analyses suggest similar or lower mucosal perforation rates and comparable short-term outcomes, while cost and operative time remain debated and likely volume-dependent. The adjunct of partial fundoplication (Dor or Toupet) continues to be standard to mitigate reflux, with comparative data showing broadly equivalent acid exposure and dysphagia control. Emerging intraoperative imaging, such as indocyanine-green near-infrared fluorescence, can assist with perfusion assessment and, in pilot reports, with confirming myotomy completeness and mucosal integrity, illustrating how evolving optics may further optimize RAHM (Figure 1) [8,20].

Indications and patient selection

RAHM is indicated for symptomatic, proven achalasia after objective confirmation with high-resolution manometry and appropriate adjunct testing, such as barium esophagram and endoscopy. Subtype stratification using the Chicago Classification v4.0 informs expectations and technique, such as longer gastric extension or tailored myotomy length, while baseline reflux risk and the option for a concomitant fundoplication are key reasons to prefer a surgical myotomy over endoscopic approaches [21,22].

Choice of RAHM versus alternatives should reflect achalasia subtype, prior interventions, anatomy, and the need for concurrent procedures. In type III disease, contemporary guidance generally favors POEM for its ability to extend a long, tailored myotomy; however, RAHM remains appropriate when surgical advantages are desired (eg, fundoplication for reflux control, hiatal hernia repair, diverticulectomy, or redo after prior POEM/LHM), guided by shared decision-making and institutional expertise. Patients with advanced/sigmoid esophagitis, obesity, or hiatal pathology may particularly benefit from the robotic platform’s visualization and suturing capabilities alongside reflux-mitigating fundoplication [21,23].

High-resolution manometry (HRM) is mandatory to confirm the achalasia subtype and guide patient counseling before RAHM. Endoscopy and/or a timed barium esophagram remain complementary tests to exclude pseudoachalasia and define the anatomy, such as a hiatal hernia or sigmoid esophagus. Baseline Eckardt scores and LES metrics should also be recorded to enable postoperative comparison. The Eckardt score is a symptom-based index for achalasia that sums four equally weighted items: dysphagia, regurgitation, chest pain, and weight loss. Each item is graded from 0 (none) to 3 (severe), yielding a total score from 0 to 12. This baseline is primarily used to monitor outcomes after intervention, where scores >3 are considered a suboptimal result [21,22,24].

Preoperative reflux assessment is useful for setting expectations and planning the antireflux strategy after myotomy. If endoscopic esophagitis is absent, ambulatory pH (± impedance) testing can establish baseline reflux burden; this also helps with shared decision-making about adding a fundoplication. Evidence syntheses suggest that adding a partial fundoplication reduces postoperative pathologic acid exposure after Heller myotomy, though the technique choice (Dor vs Toupet) is still debated [21,25,26].

Comparative efficacy and durability of RAHM, LHM, and POEM

Across contemporary series and meta-analyses, RAHM achieves high rates of clinical success with durable symptom control. Pooled comparisons of RAHM versus LHM show similar or slightly improved functional outcomes after RAHM, including reduction in Eckardt scores and dysphagia relief, with no clinically meaningful differences in need for additional therapy in the short to mid-term. In an updated meta-analysis (14 studies; >12,000 patients), RAHM achieved comparable postoperative dysphagia improvement to LHM and was associated with a lower risk of intraoperative mucosal perforation; functional outcomes were otherwise similar, supporting the efficacy of the robotic approach [8,27]. Physiologically, both RAHM and LHM produce substantial decreases in lower esophageal sphincter (LES) pressure that correlate with symptom improvement [21].

Technical and Operative Considerations

The operative characteristics differ significantly across the three techniques. POEM consistently demonstrates the shortest operative times, ranging from 101 to 121 minutes, compared with LHM (100-264) and RAHM (141-355). This efficiency advantage of POEM reflects its natural orifice approach, avoiding the need for abdominal incisions and trocar placement. However, RAHM experiences a substantial learning curve, with operative times decreasing from 163 to 113 minutes after the initial learning phase [8,28]. A critical technical advantage of POEM is its ability to achieve longer myotomies (11.6-16 cm) compared to surgical approaches (8-8.6 cm). This extended myotomy length proves particularly beneficial for type III achalasia, where spastic contractions require more extensive muscle division [28-30].

The precision advantage of RAHM is evident in significantly lower esophageal perforation rates. Multiple studies demonstrate perforation rates of 0% for RAHM compared to 11-16% for LHM. This safety profile stems from enhanced 3D visualization and improved instrument dexterity, allowing more precise tissue dissection and muscle fiber identification [8,31]. Redo myotomy after prior therapy is increasingly encountered as POEM/LHM adoption rises; 8-15% of patients develop persistent or recurrent dysphagia over time, often due to incomplete myotomy, scarring/fibrosis, twisted or disrupted fundoplication, or progressive dilation. In this context, RAHM offers three-dimensional visualization, wristed articulation, and precise dissection in scarred planes that can facilitate safe re-entry and controlled extension of the myotomy. Contemporary reviews of revisional achalasia surgery emphasize these pathologies and the need for individualized strategies, while technical expositions demonstrate stepwise robotic approaches to redo myotomy after failed Heller procedures [27,32].

Clinical Efficacy and Long-Term Outcomes

Using the Eckardt score, all three approaches demonstrate excellent clinical efficacy, with success rates ranging from 72.7% to 98% for POEM, 65.1% to 91.4% for LHM, and comparable rates for RAHM. Long-term follow-up data at four to five years show sustained efficacy, with POEM achieving 75% success and LHM 70.8% success rates. However, POEM shows particular superiority in type III achalasia management, with success rates of 53.3% versus 44.4% for Heller myotomy [28,30,33,34]. The robotic approach demonstrates potential advantages in long-term outcomes for patients with normal esophageal morphology. In matched cohorts, RAHM patients showed higher rates of complete barium emptying at four years (54% vs 34%), better Eckardt scores (1.7% vs 10% >3), and fewer reinterventions (1.2% vs 11% at three years). These findings suggest that the enhanced precision of robotic surgery may translate into superior durability of symptom relief [35].

The management of postoperative GERD represents a significant differentiating factor between techniques. POEM demonstrates consistently higher rates of postoperative reflux and erosive esophagitis compared to LHM and RHM. This difference reflects the absence of anti-reflux procedures with POEM versus the routine addition of fundoplication with surgical approaches [36,37].

RAHM demonstrates intermediate recovery characteristics, with enhanced surgeon confidence in early discharge due to reduced perforation risk. A recent series reported 12 of 13 RAHM patients discharged as day cases or with single overnight stays. Blood loss is consistently lower with RAHM (mean difference of 61.11 ml compared to LHM), contributing to improved perioperative outcomes [38].

Cost-Effectiveness Analysis

**Figure 2:**
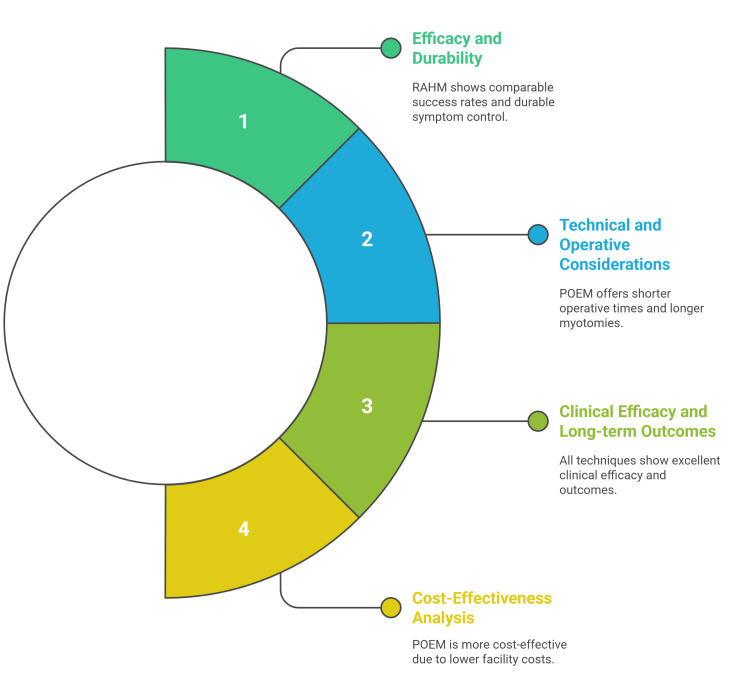
Comparative Efficacy and Durability of RAHM, LHM, and POEM RAHM: Robotic-assisted Heller myotomy, LHM: Laparoscopic Heller myotomy, POEM: Peroral endoscopic myotomy. Figure Credit: Momen Abdelglil. References: [28-40].

Economic considerations significantly influence treatment selection. POEM demonstrates superior cost-effectiveness compared to RAHM ($14,481 vs $17,782), primarily due to reduced facility costs and shorter hospital stays. The endoscopic approach can be performed in dedicated suites rather than operating rooms, substantially reducing overhead costs [39]. However, the cost-effectiveness of POEM relative to LHM shows more complex dynamics. While procedure costs may be similar ($8,630 for POEM vs $7,604 for LHM), quality-adjusted life years (QALYs) favor POEM (0.413 vs 0.357 QALYs). The incremental cost-effectiveness ratio suggests POEM costs an additional $18,536 per QALY gained, with a 68.31% probability of cost-effectiveness at a $100,000 willingness-to-pay threshold [40]. RAHM consistently demonstrates the highest costs due to robotic system expenses and longer operative times. Studies report 27% higher costs than LHM ($42,900 vs. $34,300), which limits widespread adoption despite clinical advantages (Figure 2) [38].

Current limitations and future directions

Cost and resource utilization are persistent constraints. Robotic platforms entail substantial capital and maintenance expenses, as well as longer setup/docking time and specialized staffing factors that can offset any efficiency gains and limit adoption outside high-volume centers. Single-center and multicenter series have repeatedly reported cost disadvantages for RAHM without clear improvements in length of stay or readmissions compared with LHM [35,41].

Evidence gaps complicate patient selection and benchmarking. High-quality randomized trials comparing RAHM with LHM or POEM are lacking; most datasets mix achalasia subtypes and vary in the use and type of fundoplication, making reflux and durability signals difficult to interpret against guideline-anchored standards of care. Until stronger prospective data clarify which patients derive the most value from robotics, decisions should weigh institutional expertise, costs, and the need for an antireflux procedure after surgical myotomy [21,42].

As robotic technology advances, future iterations are expected to incorporate more sophisticated visualization systems and instrument designs, which will further minimize surgical complications [43]. In a published study, the learning curve for RAHM appeared more favorable than traditional approaches, with proficiency achievable after approximately 16-18 cases compared to 19-20 cases for laparoscopic procedures [44].

The future of robotic Heller myotomy will be significantly enhanced by the integration of artificial intelligence (AI) and machine learning technologies. Current research demonstrates the potential for AI-powered intraoperative video analysis to provide real-time surgical phase recognition, anatomical landmark identification, and technical skill assessment. These systems can offer automated intraoperative guidance to support clinical decision-making and identify critical anatomical structures during some esophageal procedures [45,46]. Machine learning algorithms are being developed to enable surgical robots to learn procedures autonomously through expert demonstration and trial-and-error approaches. This represents a paradigm shift toward more autonomous surgical systems that could eventually perform complex procedures with minimal human intervention [47,48].

One of the most significant future developments in surgery will be the widespread implementation of haptic feedback systems. Current research demonstrates that haptic feedback can reduce applied forces by 83%, improve surgical accuracy by 150%, and decrease completion times by 83% during robotic surgical procedures. The Saroa pneumatic surgical robot has already shown clinical success in providing real-time haptic feedback, allowing surgeons to feel tissue resistance and prevent inadvertent organ damage [49-51].

## Conclusions

RAHM delivers symptom relief and physiologic improvement comparable to LHM, with fewer mucosal perforations. Peroral endoscopic myotomy (POEM) enables longer, tailored myotomies, especially in type III achalasia. However, it also carries a higher reflux risk without an antireflux procedure, whereas fundoplication remains standard alongside surgical myotomy. Durability across approaches is generally high. Observational data suggest potential advantages for RAHM in barium emptying, reintervention rates, and Eckardt scores in selected patients, and the platform is particularly useful in revisional cases or when concurrent foregut repairs are needed. Nonetheless, RAHM typically costs more and may require longer operative times, influencing adoption outside high-volume centers.

Future progress hinges on closing evidence gaps with prospective, subtype-specific trials that standardize antireflux strategy and long-term outcome measures. Technological advances such as enhanced visualization, AI-assisted intraoperative guidance, and haptic feedback may improve precision and safety, while competition and workflow optimization could reduce costs. Multidisciplinary, patient-centered selection should remain paramount as these innovations mature.

## References

[1] Ates F, Vaezi MF (2015). The pathogenesis and management of achalasia: current status and future directions. Gut Liver.

[2] Rogers AB, Rogers BD, Gyawali P (2020). Pathophysiology of achalasia. Annals of Esophagus.

[3] Rengarajan A, Bazarbashi AN, Gyawali CP (2025). Pathophysiology of achalasia. Digestion.

[4] Momodu II, Wallen JM (2023). Achalasia. StatPearls.

[5] Essadni Y, Serraj I, Salihoun M, Acharki M, Kabbaj N (2024). Achalasia and its subtypes: a Moroccan cohort in Ibn Sina Hospital, Rabat. PAMJ Clinical Medicine.

[6] Lee K, Hong SP, Yoo IK (2024). Global trends in incidence and prevalence of achalasia, 1925-2021: a systematic review and meta-analysis. United European Gastroenterol J.

[7] Torresan F, Ioannou A, Azzaroli F, Bazzoli F (2015). Treatment of achalasia in the era of high-resolution manometry. Ann Gastroenterol.

[8] Ataya K, Bsat A, Aljaafreh A, Bourji H, Al Ayoubi AR, Hassan N (2023). Robot-assisted heller myotomy versus laparoscopic heller myotomy: a systematic review and meta-analysis. Cureus.

[9] Perry KA, Kanji A, Drosdeck JM, Linn JG, Chan A, Muscarella P, Melvin WS (2014). Efficacy and durability of robotic Heller myotomy for achalasia: patient symptoms and satisfaction at long-term follow-up. Surg Endosc.

[10] Marano L, Pallabazzer G, Solito B (2016). Surgery or peroral esophageal myotomy for achalasia: a systematic review and meta-analysis. Medicine (Baltimore).

[11] Miller HJ, Neupane R, Fayezizadeh M, Majumder A, Marks JM (2017). POEM is a cost-effective procedure: cost-utility analysis of endoscopic and surgical treatment options in the management of achalasia. Surg Endosc.

[12] Bhayani NH, Kurian AA, Dunst CM, Sharata AM, Rieder E, Swanstrom LL (2014). A comparative study on comprehensive, objective outcomes of laparoscopic Heller myotomy with per-oral endoscopic myotomy (POEM) for achalasia. Ann Surg.

[13] Liu L, Lewis N, Mhaskar R, Sujka J, DuCoin C (2023). Robotic-assisted foregut surgery is associated with lower rates of complication and shorter post-operative length of stay. Surg Endosc.

[14] Shaligram A, Unnirevi J, Simorov A, Kothari VM, Oleynikov D (2012). How does the robot affect outcomes? A retrospective review of open, laparoscopic, and robotic Heller myotomy for achalasia. Surg Endosc.

[15] Bakhos CT, Salami AC, Kaiser LR, Petrov RV, Abbas AE (2019). Outcomes of octogenarians with esophageal cancer: an analysis of the National Cancer Database. Dis Esophagus.

[16] Rajdev PA, Hunter JG (2020). Laparoscopic cardiomyotomy: historical overview and current operative approach. Annals of Esophagus.

[17] Arcerito M, Jamal MM, Perez MG, Kaur H, Sundahl A, Moon JT (2022). Esophageal achalasia: from laparoscopic to robotic Heller myotomy and Dor fundoplication. JSLS.

[18] Rivero-Moreno Y, Echevarria S, Vidal-Valderrama C (2023). Robotic surgery: a comprehensive review of the literature and current trends. Cureus.

[19] Morrell AL, Morrell-Junior AC, Morrell AG, Mendes JM, Tustumi F, DE-Oliveira-E-Silva LG, Morrell A (2021). The history of robotic surgery and its evolution: when illusion becomes reality. Rev Col Bras Cir.

[20] Torres-Villalobos G, Coss-Adame E, Furuzawa-Carballeda J (2018). Dor Vs Toupet fundoplication after laparoscopic heller myotomy: long-term randomized controlled trial evaluated by high-resolution manometry. J Gastrointest Surg.

[21] Vaezi MF, Pandolfino JE, Yadlapati RH, Greer KB, Kavitt RT (2020). ACG clinical guidelines: diagnosis and management of achalasia. Am J Gastroenterol.

[22] Fox MR, Sweis R, Yadlapati R (2021). Chicago classification version 4.0(©) technical review: update on standard high-resolution manometry protocol for the assessment of esophageal motility. Neurogastroenterol Motil.

[23] Mustian M, Wong K (2025). Surgical management of achalasia. Abdom Radiol (NY).

[24] Yadlapati R, Kahrilas PJ, Fox MR (2021). Esophageal motility disorders on high-resolution manometry: Chicago classification version 4.0(©). Neurogastroenterol Motil.

[25] Aiolfi A, Tornese S, Bonitta G (2020). Dor versus Toupet fundoplication after laparoscopic Heller myotomy: systematic review and Bayesian meta-analysis of randomized controlled trials. Asian J Surg.

[26] Elyasinia F, Sadeghian E, Gapeleh R, Eslamian R, Najjari K, Soroush A (2022). The role of fundoplication after laparoscopic Heller myotomy in reducing postoperative symptoms in patients with achalasia: a controlled clinical trial. Middle East J Dig Dis.

[27] Aiolfi A, Damiani R, Manara M (2025). Robotic versus laparoscopic heller myotomy for esophageal achalasia: an updated systematic review and meta-analysis. Langenbecks Arch Surg.

[28] Ahmed K, Rauf SA, Hussain T (2025). Evolving therapeutic approaches in achalasia: a comprehensive review of peroral endoscopic myotomy (POEM) vs. Heller's myotomy. Ann Med Surg (Lond).

[29] Kumbhari V, Tieu AH, Onimaru M (2015). Peroral endoscopic myotomy (POEM) vs laparoscopic Heller myotomy (LHM) for the treatment of Type III achalasia in 75 patients: a multicenter comparative study. Endosc Int Open.

[30] Podboy AJ, Hwang JH, Rivas H (2021). Long-term outcomes of per-oral endoscopic myotomy compared to laparoscopic Heller myotomy for achalasia: a single-center experience. Surg Endosc.

[31] Kaaki S, Hartwig MG (2022). Robotic Heller myotomy and Dor fundoplication: twelve steps. JTCVS Tech.

[32] Cubisino A, Schlottmann F, Dreifuss NH (2022). Robotic redo Heller myotomy: how I do it?. Langenbecks Arch Surg.

[33] Fukushima N, Masuda T, Tsuboi K, Watanabe J, Yano F (2024). Long-term outcomes of treatment for achalasia: laparoscopic Heller myotomy versus POEM. Ann Gastroenterol Surg.

[34] Hugova K, Mares J, Hakanson B Per-oral endoscopic myotomy versus laparoscopic Heller’s myotomy plus Dor fundoplication in patients with idiopathic achalasia: 5-year follow-up of a multicentre, randomised, open-label, non-inferiority trial. Lancet Gastroenterol Hepatol.

[35] Raja S, Adhikari S, Blackstone EH, Toth AJ, Rice TW, Ahmad U, Murthy SC (2022). A comparative study of robotic and laparoscopic approaches to Heller myotomy. J Thorac Cardiovasc Surg.

[36] Patel K, Abbassi-Ghadi N, Markar S, Kumar S, Jethwa P, Zaninotto G (2016). Peroral endoscopic myotomy for the treatment of esophageal achalasia: systematic review and pooled analysis. Dis Esophagus.

[37] So Taa Kum A, Nunes BC, Moura ET, Franco MC, de Moura EG (2025). Gastroesophageal reflux disease over time in endoscopic versus surgical myotomy for treatment of achalasia: Systematic review and meta-analysis. Endosc Int Open.

[38] Nevins EJ, Greene K, Bawa S, Horgan L (2024). Robotic Heller's cardiomyotomy for achalasia: early outcomes for a high-volume UK centre. Ann R Coll Surg Engl.

[39] Nabi Z, Ramchandani M, Reddy DN (2017). The choice of myotomy in achalasia cardia: Heller's or per-oral endoscopic myotomy. Saudi J Gastroenterol.

[40] Greenleaf EK, Winder JS, Hollenbeak CS, Haluck RS, Mathew A, Pauli EM (2018). Cost-effectiveness of per oral endoscopic myotomy relative to laparoscopic Heller myotomy for the treatment of achalasia. Surg Endosc.

[41] Hernández IER, Kapoor H, Akimoto S, Nandipati KC, Pallati PK, Mittal SK (2015). Robotic assisted vs laparoscopic Heller myotomy: a single institution experience. J Am Coll Surg.

[42] Xie J, Vatsan MS, Gangemi A (2021). Laparoscopic versus robotic-assisted Heller myotomy for the treatment of achalasia: a systematic review with meta-analysis. Int J Med Robot.

[43] Chan EG, Sarkaria IS (2021). Robotic assisted Heller myotomy: indications, techniques and outcomes. Shanghai Chest.

[44] Jiang X, Ye C, Jiang L (2023). Single-center experience of transitioning from video-assisted laparoscopic to robotic Heller myotomy with Dor fundoplication for esophageal motility disorders. BMC Surg.

[45] Cizmic A, Mitra AT, Preukschas AA (2025). Artificial intelligence for intraoperative video analysis in robotic-assisted esophagectomy. Surg Endosc.

[46] Melvin WS, Needleman BJ, Krause KR, Wolf RK, Michler RE, Ellison EC (2001). Computer-assisted robotic heller myotomy: initial case report. J Laparoendosc Adv Surg Tech A.

[47] Qian C, Ren H (2025). Chapter 9 - Deep reinforcement learning in surgical robotics: enhancing the automation level. Handbook of Robotic Surgery.

[48] Ma R, Vanstrum EB, Lee R, Chen J, Hung AJ (2020). Machine learning in the optimization of robotics in the operative field. Curr Opin Urol.

[49] Ueda Y, Miyahara S, Tokuishi K, Nakajima H, Waseda R, Shiraishi T, Sato T (2024). First clinical application of a surgical robot with haptic force feedback function for thoracic surgery: a case report. Shanghai Chest.

[50] Bergholz M, Ferle M, Weber BM (2023). The benefits of haptic feedback in robot assisted surgery and their moderators: a meta-analysis. Sci Rep.

[51] Ueda Y, Miyahara S, Tokuishi K, Nakajima H, Waseda R, Shiraishi T, Sato T (2023). Impact of a pneumatic surgical robot with haptic feedback function on surgical manipulation. Sci Rep.

